# Aortic Calcification and Femoral Bone Density Are Independently Associated with Left Ventricular Mass in Patients with Chronic Kidney Disease

**DOI:** 10.1371/journal.pone.0039241

**Published:** 2012-06-18

**Authors:** Colin D. Chue, Nadezhda A. Wall, Nicola J. Crabtree, Daniel Zehnder, William E. Moody, Nicola C. Edwards, Richard P. Steeds, Jonathan N. Townend, Charles J. Ferro

**Affiliations:** 1 School of Clinical and Experimental Medicine, College of Medical and Dental Sciences, University of Birmingham, Birmingham, United Kingdom; 2 Department of Cardiology, Queen Elizabeth Hospital Birmingham, Birmingham, United Kingdom; 3 School of Immunity and Infection, College of Medical and Dental Sciences, University of Birmingham, Birmingham, United Kingdom; 4 Department of Endocrinology, Birmingham Children's Hospital, Birmingham, United Kingdom; 5 Clinical Sciences Research Institute, University of Warwick, Coventry, United Kingdom; 6 Department of Nephrology, Queen Elizabeth Hospital Birmingham, Birmingham, United Kingdom; Georgia Health Sciences University, United States of America

## Abstract

**Background:**

Vascular calcification and reduced bone density are prevalent in chronic kidney disease and linked to increased cardiovascular risk. The mechanism is unknown. We assessed the relationship between vascular calcification, femoral bone density and left ventricular mass in patients with stage 3 non-diabetic chronic kidney disease in a cross-sectional observational study.

**Methodology and Principal Findings:**

A total of 120 patients were recruited (54% male, mean age 55±14 years, mean glomerular filtration rate 50±13 ml/min/1.73 m^2^). Abdominal aortic calcification was assessed using lateral lumbar spine radiography and was present in 48%. Mean femoral Z-score measured using dual energy x-ray absorptiometry was 0.60±1.06. Cardiovascular magnetic resonance imaging was used to determine left ventricular mass. One patient had left ventricular hypertrophy. Subjects with aortic calcification had higher left ventricular mass compared to those without (56±16 vs. 48±12 g/m^2^, P = 0.002), as did patients with femoral Z-scores below zero (56±15 vs. 49±13 g/m^2^, P = 0.01). In univariate analysis presence of aortic calcification correlated with left ventricular mass (r = 0.32, P = 0.001); mean femoral Z-score inversely correlated with left ventricular mass (r = −0.28, P = 0.004). In a multivariate regression model that included presence of aortic calcification, mean femoral Z-score, gender and 24-hour systolic blood pressure, 46% of the variability in left ventricular mass was explained (P<0.001).

**Conclusions:**

In patients with stage 3 non-diabetic chronic kidney disease, lower mean femoral Z-score and presence of aortic calcification are independently associated with increased left ventricular mass. Further research exploring the pathophysiology that underlies these relationships is warranted.

## Introduction

Cardiovascular morbidity and mortality are increased in patients with chronic kidney disease (CKD) and have an independent inverse relationship with glomerular filtration rate (GFR) [1]. There is accumulating evidence that much of this increased risk is due to underlying structural heart disease, such as left ventricular hypertrophy (LVH), leading to arrhythmia and heart failure [2], [3]. Left ventricular mass (LVM) assessed as a continuous variable is increasingly recognised as an important prognostic marker [4], and reductions in LVM are associated with improved outcome in patients with increased cardiovascular risk [5].

Patients with CKD have deranged bone-mineral metabolism, with increased levels of the phosphaturic hormone fibroblast growth factor 23 (FGF-23) and parathyroid hormone (PTH) arising from impaired renal excretion of phosphate and impaired vitamin D activation [6]. Bone mineral density (BMD) measured using dual-energy x-ray absorptiometry (DEXA) scanning is commonly reduced in patients with CKD [7] and is an independent predictor of cardiovascular mortality in dialysis patients [8]. Vascular calcification is also highly prevalent in CKD [9] and end stage kidney disease (ESKD) [10] and is associated with increased mortality [11], [12]. The mechanism by which reduced BMD and vascular calcification might influence cardiovascular outcome in CKD is unknown. We therefore explored the relationship between femoral BMD, vascular calcification and LVM, measured using the gold-standard technique of cardiovascular magnetic resonance imaging, in a cohort of patients with stage 3 non-diabetic CKD.

## Methods

### Objectives

To determine the relationship between femoral bone density, presence of vascular calcification and LVM in patients with stage 3 non-diabetic CKD.

### Participants

Subjects were recruited from the renal clinics at the Queen Elizabeth Hospital Birmingham, UK, from 2009 to 2011. Patients were included if aged 18–80 years with stage 3 CKD (GFR 30–59 mL/min/1.73 m^2^) [13] and no change in medication in the preceding three months. Patients with heart failure, atrial fibrillation, moderate or severe valvular disease, uncontrolled hypertension (office blood pressure >140/90 mmHg), total serum cholesterol >5.5 mmol/L whether on or off treatment and diabetes mellitus were excluded. Patients receiving treatment with a phosphate binder or vitamin D analogue were also excluded.

### Data collection

Demographic data including body mass index and heart rate were recorded. Routine haematological and biochemical parameters were measured. Serum calcium levels were corrected for serum albumin. GFR was estimated (eGFR) using the four-variable Modification of Diet in Renal Disease formula with serum creatinine recalibrated to be traceable to an isotope derived mass spectroscopy method [14]. High sensitive C-reactive protein (hsCRP) was measured using a validated immunoassay (IBL International GMBH, Hamburg, Germany). Albuminuria was measured using a spot albumin: creatinine ratio. Calcium-phosphate homeostasis was assessed through measurement of the phosphatonin FGF-23, its soluble co-receptor klotho, and vitamin D. Fibroblast growth factor 23 was measured using a two-site enzyme-linked immunosorbent assay (Kainos Laboratories Inc., Tokyo, Japan). Soluble serum α-klotho was determined using a solid phase sandwich immunoassay (Immuno-Biological Laboratories Co., Japan). 1,25-dihydroxyvitamin D was measured using immunoextraction followed by quantitation by immunoassay (Immunodiagnostic Systems Ltd, Tyne and Wear, UK). 25-hydroxyvitamin D was measured by liquid chromatography-tandem mass spectrometry. Urinary fractional excretion of phosphate was calculated from 24-hour urine collections using the equation [(urine phosphate x serum creatinine)/(urine creatinine x serum phosphate)] x100, which allows correction for incomplete urine collections over 24-hours and standardises urinary phosphate excretion according to degree of renal impairment [15]. Office brachial blood pressure (BP) was recorded in the non-dominant arm in triplicate following 15 minutes of supine rest using a validated oscillometric sphygmomanometer (Dinamap ProCare 200, GE Healthcare, United Kingdom) [16] according to British Hypertension Society guidelines [17]. All subjects underwent twenty-four hour ambulatory BP measurement (Meditech ABPM-04, PMS Instruments, Maidenhead, United Kingdom). Arterial stiffness was assessed using the gold-standard measurement of carotid-femoral pulse wave velocity (PWV, SphygmoCor, AtCor Medical, Sydney) as previously described using a high fidelity micromanometer (SPC-301, Millar Instruments, Texas) to sequentially record electrocardiographically-gated carotid and femoral waveforms following 15 minutes of supine rest [18].

### Assessment of bone density

Dual energy x-ray absorptiometry scanning (Hologic QDR Series 4500 with Discovery Software version 11.02:03, Hologic Europe, Zaventem, Belgium) was used to assess BMD of both proximal femurs (femoral neck, Ward's region, trochanteric region). All scans were reported by an experienced bone densitometry clinical scientist blinded to clinical data. T-scores that refer to the young adult reference mean calculated from the manufacturer's database (based on the National Health and Nutrition Examination Survey (NHANES) population) [19] were determined. Osteopenia and osteoporosis were defined according to the World Health Organisation definitions (normal bone T-score >−1, osteopenia T-score −1 to −2.5 and osteoporosis T-score <−2.5). The BMD of each subject was standardised by calculating the difference between the observed and predicted values (sourced from the manufacturer's reference database based on the NHANES population) [19] divided by the square root of the estimated variance. This derived standard score, or Z-score, is a measure of the deviation from the expected population mean, adjusted for the covariance on a scale with zero mean and unit standard deviation, so that 95% of the normal population will have a Z-score between -2 and 2 [20]. Coefficients of variation in our clinic are 1.1%.

### Assessment of aortic calcification

The presence and extent of abdominal aortic calcification were assessed using a lateral lumbar spine radiograph and validated semi-quantitative scoring method as previously described [21]. This semi-quantitative technique shows good correlation with electron beam computed tomography assessment [22]. The abdominal aorta adjacent to the L1-L4 vertebra was divided into four sections using the midpoint of each intervertebral space as a boundary. The anterior and posterior aortic walls of each section were scored out of 3 according to the extent of calcification present with a maximum score of 24 for the entire abdominal aorta. All radiographs were analysed and scored by two independent observers. Any discordance in the presence or absence of calcification between the two observers resulted in re-assessment of that radiograph and evaluation by a radiologist to reach a final consensus.

**Table 1:** Demographic data for all subjects and according to presence or absence of abdominal aortic calcification.

	All Patients	Calcification	No Calcification	P
	(n = 120)	(n = 57)	(n = 63)	
Age	55±14	63±11	47±12	<0.001
Males (%)	65 (54)	38 (67)	27 (43)	0.01
History of cardiovascular disease (%)	13 (11)	12 (21)	1 (2)	0.002
Smokers (%)	64 (53)	38 (68)	26 (41)	0.007
Body mass index (kg/m^2^)	29.0±5.5	29.2±3.5	28.9±6.9	0.71
Office systolic blood pressure (mmHg)	128±17	133±17	123±15	0.001
Office diastolic blood pressure (mmHg)	71±11	71±12	72±10	0.61
Pulse pressure (mmHg)	57±17	62±19	52±13	<0.001
24-hour systolic blood pressure (mmHg)	124±12	127±12	121±12	0.007
24-hour diastolic blood pressure (mmHg)	71±9	70±8	72±9	0.19
24-hour pulse pressure (mmHg)	53±11	57±12	49±8	<0.001
Heart rate (bpm)	63±10	62±12	63±9	0.82

Data are mean±standard deviation or frequency (%). Analysed using unpaired t-tests or χ^2^.

**Table 2:** Renal diagnoses.

Renal Diagnosis	Frequency (%)
Hypertensive nephropathy	16 (13)
IgA nephropathy	13 (11)
Reflux nephropathy	12 (10)
Adult polycystic kidney disease	12 (10)
Systemic vasculitis	13 (11)
Focal segmental glomerulosclerosis	8 (7)
Nephrectomy for neoplasm	5 (4)
Other glomerulonephritis	7 (6)
Other	30 (25)
Unknown	4 (3)

Data are frequency (percentage).

**Table 3:** Medication use.

Medication	Frequency (%)
ACEI	64 (53)
ARB	32 (27)
ACEI and/or ARB	92 (77)
Aspirin	23 (19)
Statin	53 (44)
Other lipid lowering therapy	6 (6)
β-blocker	20 (17)
Calcium-channel blocker	33 (28)
Diuretic	32 (27)
α-blocker	17 (14)
Calcium supplement	8 (7)
Bisphosphonate	9 (8)
Prednisolone	18 (15)
Immunosuppressant	23 (19)

Data are frequency (percentage). ACEI, angiotensin converting enzyme inhibitor; ARB, angiotensin receptor blocker.

**Table 4:** Biochemical data for all subjects and according to presence or absence of abdominal aortic calcification.

	All Patients	Calcification	No Calcification	P
	(n = 120)	(n = 57)	(n = 63)	
eGFR (mL/min/1.73m^2^)	50±13	48±13	52±13	0.09
Total cholesterol (mmol/L)	4.9±1.2	4.8±1.1	5.0±1.3	0.32
Triglycerides (mmol/L)	1.16 (0.84–2.00)	1.39 (0.98–2.15)	0.98 (0.73–1.40)	0.01
hsCRP (μg/mL)	2.21 (1.15–6.37)	3.02 (1.13–8.52)	1.96 (1.15–5.96)	0.54
Phosphate (mmol/L)	1.03±0.16	1.03±0.17	1.03±0.17	0.98
Phosphate >1.4mmol/L (%)	4 (3)	2 (4)	2 (3)	1.00
Calcium (mmol/L)	2.21±0.09	2.23±0.09	2.19±0.10	0.04
Parathyroid hormone (ng/L)^a^	52 (38–71)	55 (42–72)	47 (35–71)	0.14
Parathyroid hormone >65ng/L (%)^a^	36 (32)	18 (36)	18 (29)	0.54
Alkaline phosphatase (mmol/L)	182±56	188±58	177±55	0.25
Fibroblast growth factor 23 (pg/mL)	67.6 (50.3–85.2)	67.0 (52.8–86.2)	67.6 (45.7–83.3)	0.34
Klotho (pg/mL)	943 ± 396	951 ± 471	936 ± 317	0.84
1,25-dihydroxyvitamin D (pmol/L)	76.0 ± 31.0	74.3 ± 26.9	77.6 ± 34.5	0.57
25-hydroxyvitamin D (nmol/L)	55.6 ± 30.5	53.9 ± 29.5	57.2 ± 31.5	0.56
Albumin: creatinine ratio (mg/mmol)	6 (1–48)	8 (1–43)	3 (1–53)	0.86
Urinary fractional excretion of phosphate	21 (13–32)	19 (14–29)	21 (12–34)	0.74

a Data available for 112 subjects. Data are mean±standard deviation, frequency (%) or median (interquartile range). Analysed using unpaired t-tests or χ^2^. eGFR, estimated glomerular filtration rate; hsCRP, high sensitive C-reactive protein.

**Table 5:** Arterial stiffness, left ventricular mass and bone mineral density data for all subjects and according to presence or absence of abdominal aortic calcification.

	All Patients	Calcification	No Calcification	P
	(n = 120)	(n = 57)	(n = 63)	
Pulse wave velocity (m/s)	8.9 (7.4–11.2)	10.0 (8.3–12.9)	8.0 (6.7–9.4)	<0.001
Pulse wave velocity_adj_ (m/s)	9.1 (7.9–10.8)	10.4 (8.6–12.9)	8.2 (6.8–9.5)	<0.001
Left ventricular ejection fraction (%)	74±7	75±8	74±6	0.20
Left ventricular mass (g)	101±30	111±30	93±28	0.001
Left ventricular mass index (g/m^2^)	52±13	57±14	48±11	0.001
Mean femoral bone mineral density (g/cm^2^)	1.00±0.14	1.02±0.14	0.98±0.13	0.20
Mean femoral T-score	0.03±0.96	0.04±0.95	0.02±0.98	0.89
Mean femoral Z-score	0.60±1.06	0.77±1.04	0.45±1.06	0.10

Data are mean±standard deviation or median (interquartile range). Analysed using unpaired t-tests.

### Cardiovascular magnetic resonance imaging

Cardiovascular magnetic resonance imaging was performed on a 1.5 Tesla scanner (Symphony, Siemens, Erlangen, Germany). Serial contiguous short axis cines were piloted from the vertical long axis and horizontal long axis of the left ventricle (electrocardiographically-gated, steady-state free precession imaging [True-FISP]; temporal resolution 40–50 ms, repetition time 3.2 ms, echo time 1.6 ms, flip angle 60°, slice thickness 7 mm) in accordance with previously validated methodologies [23]. Analysis was performed offline (Argus Software, Siemens, Erlangen, Germany) by a single observer for measurement of left ventricular function, volumes and LVM [23], which was indexed to body surface area (LVMI; Mosteller formula, BSA (m^2^) =  √((weight (kg) × height (cm))/3600). Left ventricular hypertrophy was defined as an LVMI greater than age and gender corrected limits [23].

### Ethics

West Midlands Research Ethics Committee approved the study and written informed consent was obtained from each participant. The study was conducted in accordance with the *Declaration of Helsinki* and the principles of *Good Clinical Practice*.

### Statistical methods

All data were analysed using SPSS version 19 (SPSS Inc, Chicago, Il, USA). Data are shown as mean±standard deviation, frequency (percentage) or median (interquartile range). Data distribution was tested using the Kolmogorov-Smirnov test and normality plots. Normally distributed variables were analysed using unpaired t-tests and χ^2^. Variables not normally distributed were log transformed prior to analysis (serum triglycerides, hsCRP, PTH, FGF-23, albumin-creatinine ratio, urinary fractional excretion of phosphate and PWV). Analysis of variance with *post hoc* Tukey was conducted to assess the impact of mean hip Z-score and presence of vascular calcification on LVMI. Pearson correlation and linear regression were used to assess the relationship between two continuous variables. Colinearity between variables was assessed by examining the variance inflation factor; a value >5 indicated colinearity. A Type I error rate below 5% (P<0.05) was considered statistically significant.

### Reproducibility

Intraobserver variability for LVMI was determined by random selection of twelve scans (10% of the cohort) for repeat analysis by the same observer. Interobserver variability for aortic calcification score was determined by comparing calcification scores between two observers for all 120 subjects. Variability of LVMI and aortic calcification score was determined using an intraclass correlation coefficient and two-way random effects model.

## Results

### Participants

Of the 1297 patients with non-diabetic CKD screened in the outpatient renal clinics, 602 met the study entry criteria. In total 494 patients were approached to take part in the CRIB-PHOS study; 374 declined to participate and therefore 120 were recruited. Demographic data for these 120 subjects are shown in Table 1. The mean age of all participants was 55±14 years with 54% male. Renal diagnoses and medication use are detailed in Tables 2 and 3. Patients were on a mean of 1.7±1.2 antihypertensive agents with 77% receiving either an angiotensin converting enzyme inhibitor and/or an angiotensin receptor blocker. Biochemical data are shown in Table 4. Three per cent of patients had a serum phosphate above 1.4 mmol/L, the upper limit of normal for our assay. Thirty-six patients (32%) had a PTH value above the upper limit of normal for our assay (65 ng/L) and two had a PTH above twice the upper limit of normal. No patients had a serum calcium concentration greater than 2.60 mmol/L. Pulse wave velocity, left ventricular mass and bone density data are shown in Table 5. Ten patients (8%) had osteopenia, of which six were over the age of 50 years; no patients were overtly osteoporotic. Ten patients (8%) had a Z-score of less than −1; mean Z-score was 0.60±1.06. Almost half (48%) of all participants had detectable calcification of the aorta, with a median calcification score of 4 (range 1–18). Only one patient (1%) had LVH.

**Figure 1:**
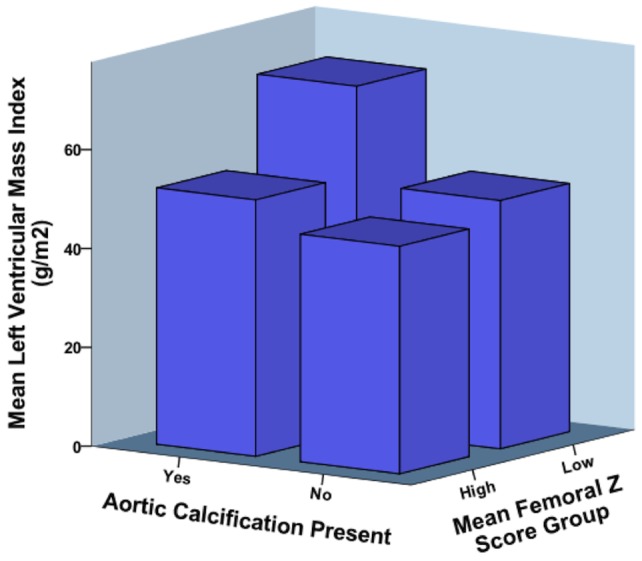
The relationship between aortic calcification, mean femoral Z-scores and left ventricular mass index. Both low mean femoral Z-score (below zero) and presence of aortic calcification were significantly associated with increased left ventricular mass index (P<0.001, two-way analysis of variance).

**Figure 2:**
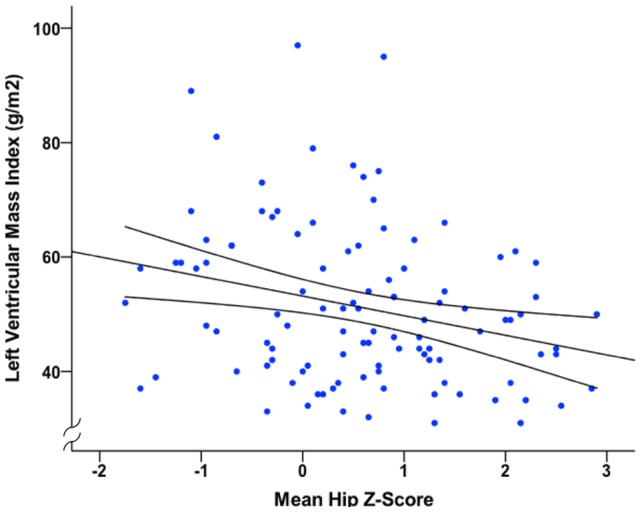
The relationship between mean femoral Z-score and left ventricular mass index. Data analysed using Pearson correlation (r^2^ = 0.072, P = 0.006).

### Aortic calcification

A comparison of demographics between patients with calcification and those without is shown in Table 1. Those with calcification were significantly older, more likely to be male and more likely to have a history of cardiovascular disease and smoking compared to those without. The only biochemical differences were higher serum triglycerides and higher serum calcium concentrations in patients with aortic calcification; there was no difference in serum phosphate, total cholesterol, hsCRP or eGFR (Table 4). There were also no significant differences in FGF-23, klotho or vitamin D. Individuals with calcification had stiffer arteries with significantly higher PWV (10.0 (8.3–12.9) vs. 8.0 (6.7–9.4) m/s, P<0.001) compared to those without (Table 5). Mean LVMI was also higher in patients with calcification (57±14 vs. 48±11 g/m^2^, P = 0.001). There was no significant difference in parameters of femoral bone density between the groups.

### Effects of bone density and vascular calcification on left ventricular mass

Subjects were divided into two groups of high or low mean femoral Z-score according to whether their mean femoral Z-score was above or below zero (the standardised mean for the population). Patients in the high mean femoral Z-score group were older (58±12 vs. 45±13 years, P<0.001), heavier (mean weight 87±15 vs. 74±14 kg, P<0.001) and had significantly lower LVMI than those in the low Z-score group (50±12 vs. 57±15 g/m^2^, P = 0.02). There were no other demographic or biochemical differences between the groups; in particular there were no significant differences in markers of bone metabolism or inflammation. A two-way analysis of variance was used to explore the effect of high or low Z-scores and the presence or absence of aortic calcification on LVMI. Both Z-score grouping and presence of aortic calcification had significant effects on LVMI (P<0.001; Figure 1).

### Correlates of left ventricular mass

In univariate analyses mean femoral Z-score and presence of aortic calcification correlated with LVMI (r = −0.28, P = 0.004 and r = 0.32, P = 0.001 respectively; Figure 2 and Table 6). All variables that significantly correlated with LVMI in univariate analyses were entered into a multiple regression model (Table 6), with 24-hour systolic BP used as the most strongly correlated variable for BP. The model explained 46% of the variability in LVMI (P<0.001). Gender, 24-hour systolic BP, presence of aortic calcification and mean femoral Z-score emerged as independent predictors of LVMI, with systolic BP, presence of calcification and mean femoral Z-score each contributing to 4% of the variation in LVMI. Substitution of 24-hour systolic BP with office systolic BP made no appreciable difference to the model.

### Reproducibility

Intraobserver variability for LVMI was excellent with an intraclass correlation coefficient of 0.992 (0.981–0.997, P<0.001). Interobserver variability for aortic calcification was also low with an intraclass correlation coefficient of 0.917 (0.883–0.941, P<0.001).

## Discussion

In this cross-sectional observational study of patients with stage 3 non-diabetic CKD a significant independent inverse relationship between femoral bone density Z-scores and LVMI was demonstrated. Furthermore, the presence of aortic calcification was associated with increased aortic stiffness and significantly greater LVMI independent of bone density. Our findings highlight important links between bone disease, vascular calcification and LVM, a prognostically important cardiovascular variable.

Several population studies have linked reduced bone density and osteoporotic fractures with cardiovascular disease, especially heart failure, although the mechanisms are unknown [24], [25],[26],[27]. Our findings suggest that underlying structural heart disease may be an important link. The presence of LVH as a marker of increased cardiovascular risk is well accepted, but the value of LVM as a continuous variable that has a graded relationship with cardiovascular risk is being increasingly recognised [4], [28]. Only one other study to date has demonstrated an association between BMD and LVM. This cross-sectional study of 460 healthy individuals showed BMD to be an independent predictor of echocardiographically-derived LVMI in post-menopausal women but not in men [29].

**Table 6:** Univariate and multivariate analyses with left ventricular mass index as the outcome variable.

	Univariate		Multivariate	
	r	P	B	95% CI
Gender	−0.50	<0.001	−0.34	−13.45–−4.48
Office systolic blood pressure (mmHg)^a^	0.31	0.001		
24-hour systolic blood pressure (mmHg)	0.40	<0.001	0.23	0.07–0.43
24-hour pulse pressure (mmHg)^a^	0.30	0.002		
Parathyroid hormone (ng/L)	0.23	0.019	0.09	−4.66–17.37
Albumin: creatinine ratio (mg/mmol)	0.25	0.01	0.12	−0.54–3.43
Presence of aortic calcification	0.32	0.001	0.21	0.89–10.12
Mean femoral Z-score	−0.28	0.004	−0.23	−4.75–−0.85

a 24-hour systolic blood pressure was used as the strongest correlating variable representing blood pressure in the model. Data analysed using Pearson correlation (univariate) and enter linear regression (multivariate). All variables that correlated significantly with left ventricular mass index in univariate analysis were entered into the multivariate regression model. R^2^ for model = 0.46, P<0.001. For gender 0 = male, 1 = female. β, standardised coefficient; 95% CI, 95% confidence interval.

Coronary artery calcification independently correlated with LVMI in a study of 118 haemodialysis patients [30]. Most of the patients in this study (75%) had established LVH on echocardiography. Our data extends these findings to include patients with early stage CKD, almost all of which had normal LVM. Vascular calcification has been shown to be a predictor of adverse cardiovascular outcome in several populations [11], [12]. This has often been attributed to a high atherosclerotic burden. Our data also supports a contributory role for increased LVM.

Arterial stiffness is a powerful predictor of poor cardiovascular outcome [12] and a number of studies, including our own, have shown a relationship between vascular calcification and increased arterial stiffness in CKD [31] and ESKD patients [32], [33], [34]. Increased arterial stiffness is characterised by augmented systolic BP and pulse pressure, which can increase afterload on the left ventricle, thus promoting ventricular stiffening, hypertrophy and fibrosis [3]. Despite higher PWV in patients with calcification, we did not demonstrate an association between arterial stiffness and LVM. A relationship between arterial stiffness and LVMI has previously been demonstrated in ESKD patients [35], but this is not a consistent finding [36]. The near-normal PWV and LVM of our cohort may have masked any potential underlying relationship between these two variables.

Several reports from the general population have demonstrated an inverse association between severity of vascular calcification and bone density [37], [38], [39], with a similar relationship also being reported in dialysis patients using coronary artery calcification scoring [40], [41]. Evidence of such a relationship in the early stage CKD population is limited. In a cross-sectional analysis of 48 patients with CKD (mean GFR 35±10 mL/min/1.73 m^2^), Toussaint *et al* demonstrated an inverse correlation between femoral arterial calcification (but not aortic calcification) assessed using computed tomography (CT) and femoral BMD measured using DEXA scanning [31]. Filgueira *et al* divided 72 patients with stage 2–4 CKD into tertiles of vertebral bone density measured using CT and found those in the lowest bone density tertile to have significantly higher coronary artery calcification than those in the other tertiles [42]. In our study we did not demonstrate a relationship between bone density and presence or extent of vascular calcification. These differences may be explained by our exclusion of diabetic patients, the relative absence of established renal bone disease and relatively preserved kidney function in our cohort (mean GFR 50±13 mL/min/1.73 m^2^) together with our use of a less sensitive semi-quantitative technique for detection of vascular calcification.

Abnormalities of mineral metabolism, such as elevated serum phosphate and calcium-phosphate product, have been identified as risk factors for the presence or progression of vascular calcification in CKD patients in a number of studies [41], but this finding is not consistent [9], [31]. Similarly FGF-23 has been linked to calcification of peripheral arteries but not of the aorta [43]. Serum calcium and triglycerides were higher amongst patients with calcification in our cohort but there were no significant differences in serum phosphate, PTH, FGF-23, klotho, vitamin D, cholesterol or alkaline phosphatase between groups. Our study does not support a role for FGF-23, klotho or vitamin D as a mediator of increased LVM in the setting of vascular calcification and reduced bone density, but this requires confirmation with larger numbers of patients. Serum levels of calcium and phosphate are poor markers of total body calcium and phosphate content, and the prevalence of hyperphosphataemia amongst our cohort was low, most likely reflecting relatively preserved kidney function.

### Limitations

Our study was observational and cross-sectional in design and thus subject to potential confounding from missing variables. We can only report associations and not infer causality. The use of lateral lumbar spine radiography and a semi-quantitative scoring method to assess severity of aortic calcification is not as sensitive as other modalities such as CT scanning and is therefore likely to have underestimated the prevalence of vascular calcification in our cohort. Nevertheless we were still able to demonstrate an independent relationship between severity of vascular calcification and LVM using this method.

### Conclusion

In patients with stage 3 non-diabetic CKD lower femoral bone density Z-scores and presence of vascular calcification are independently associated with increased LVM. Further studies into the mechanisms and potential effect of treatments that maintain bone density and attenuate progression of vascular calcification on LVM are warranted.
